# Two Cases of Leigh Syndrome in One Family: Diagnostic Challenges and Clinical Management Experience in Latvia

**DOI:** 10.1155/2021/5266820

**Published:** 2021-11-26

**Authors:** Arta Katkevica, Madara Kreile, Ieva Grinfelde, Gita Taurina, Ieva Micule, Iveta Dzivite-Krisane, Arta Smite-Laguna, Ieva Malniece

**Affiliations:** ^1^Riga Stradiņš University, Riga, Latvia; ^2^Childen's Clinical University Hospital, Riga, Latvia

## Abstract

Leigh syndrome is a neurodegenerative disorder with an incidence of 1 : 40,000 live births. The clinical presentation of LS is highly variable with heterogeneity in the disease-associated symptoms of cerebellar, motor, and extrapyramidal dysfunction and common infections. There is no effective treatment for this condition; as such, the prognosis of this condition is very poor with death occurring within the first few years of life. In this study, we report the first LS case in Latvia with SURF1 pathogenic variants in two siblings. The difficulties encountered establishing a diagnosis for the first proband and the effective prenatal diagnosis for the second offspring that led to termination of the pregnancy are discussed.

## 1. Introduction and Definition

Leigh syndrome (LS), also referred to as subacute necrotising encephalomyelopathy, was ﬁrst described by a British psychiatrist and neuropathologist, Denis Archibald Leigh, in 1951 [1]. LS may be associated with deficiency—either isolated or combined—of any oxidative phosphorylation (OXPHOS) enzyme. Underlying causes can be found in the nuclear DNA or mitochondrial DNA [2, 3]. Coenzyme Q10 deﬁciency and disturbed pyruvate metabolism are also known causes of LS. The clinical presentation of LS is highly variable with heterogeneity in the disease-associated symptoms of cerebellar, motor, and extrapyramidal dysfunction and common infections [4]. However, in most cases, it presents as a progressive neurological disease with motor and intellectual developmental delay and signs and symptoms of brainstem and/or basal ganglia involvement [5–7]. Brainstem dysfunction is the most frequently observed clinical manifestation of the disease.

The phenotypic heterogeneity of LS makes its diagnosis complicated, which is generally established through biochemical, radiological, and genetic evaluation followed by histological evaluation of the patient [3, 5, 6].

LS is the most common paediatric mitochondrial disorder with an estimated global prevalence of 1 : 40,000 live births, although it has a relatively higher or lower prevalence in certain populations [3]. With a population of approximately 1.9 million (latest data compiled by the Central Statistical Bureau of Latvia, 2019), Latvia yields a small number of LS patients. In this case report, we present the first molecularly confirmed case of *SURF1* LS in Latvia. Our report details the clinical presentation, the investigation process leading to confirmation with particular emphasis on the underlying genetic heterogeneity, and the available treatment options.

## 2. Case Description

A 22-month-old male child was referred to Children's Clinical University Hospital in Riga from another hospital due to an episode of hypoglycaemia (1.6 mmol/L). His general health condition was evaluated as serious. He was the second child of nonconsanguineous parents and was born at term after an uneventful pregnancy. Physical and psychomotor development was documented to be delayed.

Laboratory findings showed metabolic crisis—ketotic hypoglycaemia, abnormal acid-base balance, increased anion gap, lactic acidaemia 5.11 mmol/L (reference range: <2.5 mmol/L), and lactic aciduria 4993 mmol/L (reference range: 0–200 mmol/L). Physical examination detected a rash on the patient's face and body, oedema of the face and both lower limbs, generalised low muscle tone, and hepatomegaly (+3 cm below the costal margin). His weight was 12 kg, and his height was 77 cm (−3 SD). Neurological investigation disclosed severe hypotonia, ataxia, and muscle hypotrophy.

The patient had feeding difficulties due to aphthous ulcers in the oral cavity. As his health condition was considered serious, the boy was admitted to the intensive care unit where he developed a high-degree fever. Several blood samples were taken for microbiological diagnostics, and culture results revealed the presence of Gram-positive flora. Fluctuating elevated lactate levels were also detected in his blood: 5.11 mmol/L initially, then 3.29 mmol/L and 2.88 mmol/L (reference range: <2.5 mmol/L).

The patient subsequently vomited haematin several times and passed dark stools. Brain magnetic resonance imaging (MRI) showed slightly wider lateral ventricles and subarachnoid space; however, no specific changes were uncovered. Brain magnetic resonance spectroscopy (MRS) showed no significant deviations in metabolites.

X-ray of the lungs and abdomen and fibrogastroscopy of the gastrointestinal tract showed no abnormal pathology.

Due to the low free carnitine and elevated lactate levels in his blood, treatment with L-carnitine (100 mg/kg/day) and thiamine (150 mg/day) supplementations was initiated as an empiric therapy for an undiagnosed mitochondrial disease.

The plasma amino acid panel showed low glutamine, glycine, serine, and citrulline levels and slightly elevated alanine and glycine levels. The organic acid spectrum in urine showed high glutaric acid, 7.13 (normal: 0–5.3). Based on the biochemical findings, a diagnosis of glutaric aciduria type 1 was suspected and, consequently, the patient's DNA was sent for sequencing analysis using a next-generation sequencing panel of 435 genes associated with metabolic disorders. The patient's clinical condition significantly improved, and he was discharged from the hospital. Nonetheless, genetic follow-up and maintenance therapy with L-carnitine and thiamine were recommended until a definitive diagnosis could be made.

After discharge, the DNA test results revealed no mutation in the gene causing glutaric aciduria type 1 and did not uncover any known disease-causing genetic pathogenic variant in the tested genes. Mitochondrial DNA sequencing was subsequently performed, but, again, no pathological variants were detected.

Nine months later, the patient was readmitted to our hospital due to lethargy, fatigue, and ataxia after withdrawal of his prescribed medications L-carnitine and thiamine. Brain MRI was repeated and showed progressive structural changes and lesions in the medulla oblongata and mesencephalon although MRS was not repeated.

A further extended genetic investigation was initiated. Specifically, whole-exome sequencing was performed to identify causative variants. Two pathogenic variants in the *SURF1* gene were identified: c.845_846del, p.(Ser282Cysfs ∗ 9) (maternal), and c.752-1G > *C* (paternal). Based on these findings and the clinical presentation, a diagnosis of LS was established. The L-carnitine/thiamine therapy was resumed. Furthermore, riboflavin and coenzyme Q10 were added to the treatment regimen. Neurological symptoms regressed for a short period of time. The child is currently four years old and is slowly deteriorating. He has lost the ability to walk and requires support to sit.

In 2020, the patient's mother was reported to be pregnant with a gestational age of 21 weeks +5 days. Foetal ultrasound at 21 + 3 weeks revealed a cerebral periventricular cyst with dimensions of 21 mm × 11 mm. Diagnostic amniocentesis was performed, and foetal DNA sequencing results revealed two pathogenic variants in the *SURF1* gene in a compound heterozygous state. Taken together, these findings were interpreted as LS and the pregnancy was terminated.

## 3. Discussion

This report details the first *SURF1*-associated LS family involving a child and a foetus in Latvia, highlighting the difficulties encountered in establishing a diagnosis in postnatal and prenatal cases.

Loss-of-function mutations in the *SURF1* gene are associated with mitochondrial complex IV deficiency, nuclear type 1, which manifests as LS.

This is an autosomal recessive metabolic disorder characterised by rapidly progressive neurodegeneration and encephalopathy with loss of motor and cognitive skills, often triggered by an infection, between about five and 18 months of age after previously normal development [8]. Our patient was 22 months old when he was referred to our hospital. His psychomotor development was assessed as delayed at presentation; presumably, his parents had not recognised prior indications of this. For the majority of *SURF1* patients, the median age for the onset of first symptoms is 9.5 months and the most frequently noted initial symptoms are poor feeding/vomiting (frequently attributed to gastro-oesophageal reflux) and poor weight gain [9].

Mutations leading to the disruption of complex IV are the most common cause of LS in Europe [9]. The causative variant c.845_846delCT in the *SURF1* gene has been reported as the most common variant in European populations, and the variant c.752-1G > *C* has been reported in one LS family of Polish ethnicity [10, 11]. However, these two mutations have never before been reported in one patient.

Most patients with pathogenic variants in *SURF1* have bilateral brain MRI abnormalities involving the brainstem and subthalamic nuclei [12]. Nevertheless, there is still a degree of variability in their brain MRI findings [9]. The radiological features of our patient were not typical for LS. He had a normal brain MRI at 22 months of age, despite a severe clinical presentation. By contrast, most of the patients reported to date exhibited some neuroradiological changes by this age and altered clinical status [4, 6, 13–15]. It was only after our patient's second hospitalisation, at 31 months of age, that the repeated brain MRI showed progressive structural changes and lesions in the medulla oblongata and mesencephalon. However, there were no alterations in subthalamic nuclei, the typical lesion localisation in patients with causative variants in *SURF1*.

Occasionally, LS-*SURF1* patients may display atypical neuroimaging features such as diffuse supratentorial leukodystrophy, unifocal or multifocal infarctions, diffuse or focal cortical atrophy, or predominant cerebellar atrophy [15]. MRI is a key diagnostic tool in LS, but the clinical spectrum of *SURF1* mutations is larger than previously thought and normal MRI findings, especially in early childhood, do not necessarily exclude *SURF1* defects. The reasons for this heterogeneity in neuroimaging whilst retaining a phenotypic presentation in LS are not well understood. Having said that, considering that LS is the result of a number of different biochemical and genetic defects that affect mitochondrial function, one would expect to find some variability in the MRI findings amongst patients. In addition to MRI, magnetic resonance spectroscopy (MRS) could be performed for patients with LS. Lactate peaks in the brain (significant peak at >1.35 ppm) or the CSF is a trademark of mitochondrial disease, and MRS detection of CNS lactate sometimes may be more sensitive than serum lactate measurement [16]. In our case, lactate was not found in areas of the brain that appeared normal on MR images and the MRS investigation was not repeated.

The biochemical presentation of LS can include elevated lactate levels in blood and/or cerebrospinal fluid and may indicate metabolic acidosis and hypoglycaemia in decompensation status. From previous reports, the increased lactate level in *SURF1* patients ranges between 2.3 (reference range <2.5 mmol/L) and 7.3 mmol/L (mean 4.4 mmol/L) [9]. Tissue hypoperfusion is the most common cause of lactate elevation; however, this is not particularly distinctive of LS as many other aetiological factors exist and many other metabolic diseases demonstrate increased lactate [17]. Our patient showed variable results in serial lactate measurements, leading us to suspect a septic aetiology of his lactacidaemia. If lactic acidosis is present because of OXPHOS defects, parallel elevations in plasma alanine are commonly observed [3, 18, 19]. Therefore, profiling of blood amino acids and urinary organic acids would be worthwhile and may provide pointers to the underlying cause.

In many suspected or unclear cases, the first genetic approach is next-generation sequencing—gene panels, clinical exome sequencing, whole-exome/genome sequencing. In our case, we performed next-generation sequencing with a panel consisting of 435 genes associated with metabolic disorders provided by a private commercial company. However, our approach underscores that clinicians should pay special attention to the contents of conventional next-generation sequencing panels. The *SURF1* gene was not included in this analysis, and so the appropriate diagnosis of our patient was delayed for an extended period of time [20]. In paediatric patients with progressive symptoms, achieving a correct diagnosis in a short period of time is still challenging.

The aim of prenatal diagnostic testing for genetic diseases is to identify causative variants, evaluate the general clinical condition of a foetus, and provide an assessment of the risk to the foetus in developing the disease antenatally or in childhood. Prenatal testing is usually necessary and requested as a consequence of having a previously affected child. The most common abnormal foetal ultrasound findings associated with mitochondrial disorders include periventricular cysts, intestinal pseudo-obstruction, and skin oedema with reduced foetal movements [19, 21]. Periventricular cysts may be the initial *in utero* presentation in patients with mitochondrial disorders, although when a nonspecific abnormal foetal ultrasound finding is detected, it might be the only feature to suggest a mitochondrial disorder [22]. The interpretation of prenatal testing and prognostic variants with regard to whether or not the child will be affected is relatively clear in those families carrying causative variants in nuclear DNA where mostly classic Mendelian rules of inheritance apply. All families affected by a mitochondrial disease should also be made aware of all the available reproductive options and alternative options for future pregnancies. Such options include gamete (egg or sperm) donation or adoption as an alternative to pregnancy. Techniques to reduce or prevent the transmission of mitochondrial DNA and nuclear DNA mutations, such as pre-implantation genetic diagnosis, are already available [21]. In essence, we stress the importance of prenatal diagnostic testing of the foetus as the first step in diagnosing LS.

*SURF1*-deficient LS has been reported to have a more positive survival outcome compared with LS associated with complex I deficiency or *LRPPRC* mutations [9]. Predictors of poorer prognosis include disease onset before six months of age, failure to thrive, brainstem lesions on neuroimaging, epileptic seizures that are difficult to treat, and a history of intensive care hospitalisation.

Generally, specific therapy for mitochondrial disorders is not widely available and no specific treatment for LS exists. The aim of symptomatic treatment is to manage symptoms and to improve the energy state by increasing and optimising ATP production, improving the efficiency of mitochondria and lowering lactate levels [20]. The use of coenzyme Q10, thiamine, and L-carnitine may decrease symptom severity in a small number of cases [20]. Supplementation with group B vitamins, such as thiamine, which is a cofactor for pyruvate dehydrogenase, had a significant effect and led to neurological improvement in our case. However, there are only a few reported cases of thiamine-responsive pyruvate dehydrogenase complex-deficient LS in the literature [23, 24]. Thiamine responsiveness is more likely in patients presenting after 12 months of age than in those presenting with neonatal lactic acidosis and corpus callosum abnormalities [24]. Based on our experience, it is important to start a therapeutic trial of thiamine immediately if metabolic disease is suspected since biotin-thiamine-responsive basal ganglia disease can fully imitate the clinical presentation of LS.

In patients suffering from LS, a few case reports have shown promising results for a treatment with EPI-743 [25, 26]. This medication is supposed to enhance the biosynthesis of glutathione and to improve oxidative stress status, whereas lactate values remain unchanged [27]. Enns et al. reported that patients with SURF1 defect had been demonstrating clinical improvement after starting EPI-743 treatment without side effects [25]. Even though the results are promising, a single case is not sufficient for assessing the therapeutic effect of a currently unapproved drug. Drug effects need to be evaluated by randomised, double-blind, placebo-controlled trials.

Despite making major progress in our understanding of the molecular mechanisms underlying mitochondrial diseases, the available therapeutic options are very limited. Thus, they remain a fundamental goal of the current research in this field. A patient's quality of life can improve significantly if appropriate physiotherapy and pharmacotherapy for neuromuscular concerns are provided. Suitable feeding methods to prevent malnutrition and aspiration and treatment of fever and infections are further important approaches to improve the quality of life and reduce the stress of the patient and their family. Cardiology, pulmonology, and ophthalmology evaluations are valuable to identify the involvement of these organs at an early stage.

## 4. Conclusions

This report details the first LS case in Latvia with *SURF1* pathogenic variants in two siblings. The difficulties encountered while establishing a diagnosis for the first proband and the effective prenatal diagnosis for the second offspring that led to termination of the pregnancy are discussed. Identifying an underlying genetic cause in LS patients can be challenging. However, it can be facilitated if clinical, biochemical, and radiological findings are carefully evaluated. LS is a rare condition, and many other conditions present with similar symptoms. Nevertheless, a diagnosis of LS should be considered for a child with delayed psychomotor development, weakness, eating difficulties, signs of gradual neurodevelopmental regression, and symptoms of brainstem and/or basal ganglia involvement, especially when the clinical deterioration and metabolic crisis coincide with intercurrent infections.

## Data Availability

The clinical data used in this case report are presented in this article.
